# Spoilage Bacteria Counts on Broiler Meat at Different Stages of Commercial Poultry Processing Plants That Use Peracetic Acid

**DOI:** 10.3390/ani12111439

**Published:** 2022-06-02

**Authors:** Hudson T. Thames, Courtney A. Fancher, Mary G. Colvin, Mika McAnally, Emily Tucker, Li Zhang, Aaron S. Kiess, Thu T. N. Dinh, Anuraj T. Sukumaran

**Affiliations:** Department of Poultry Science, Mississippi State University, Mississippi State, MS 39762, USA; htt37@msstate.edu (H.T.T.); caf232@msstate.edu (C.A.F.); mgc139@msstate.edu (M.G.C.); mfm219@msstate.edu (M.M.); ert138@msstate.edu (E.T.); lz245@msstate.edu (L.Z.); askiess@ncsu.edu (A.S.K.); thu.dinh@msstate.edu (T.T.N.D.)

**Keywords:** spoilage, peracetic acid, poultry processing, prevalence, broiler

## Abstract

**Simple Summary:**

The presence of spoilage bacteria on broiler meat is known to cause deleterious effects, such as off odors and color, and contributes to a shorter shelf life. While spoilage microbes do not pose a major health concern, the presence of these bacteria results in economic loss that could be mitigated during processing. In order to evaluate the efficacy of antimicrobial interventions on spoilage microbes, we collected broiler meat samples from various stages of commercial poultry processing plants that use different applications and concentrations of peracetic acid. From these results, we conclude that the most effective intervention occurs during carcass chilling. However, the presence of aerobic bacteria and coliforms on broiler meat during second processing suggests cross contamination, which could affect the shelf life of retail chicken parts.

**Abstract:**

In poultry processing, spoilage microbes are persistent microorganisms, which affect the quality of broiler meat. Peracetic acid (PAA) is the most common antimicrobial used by commercial processing plants, which can reduce the prevalence of these microbes. The goal of this study was to determine the concentrations of aerobic bacteria, coliforms, lactic acid bacteria, and *Pseudomonas* on broiler meat in processing plants that use peracetic acid in various concentrations as the primary antimicrobial. Samples were collected from three processing plants at five processing steps: post-pick (defeathering), pre-chill, post-chill, mechanically deboned meat (MDM), and drumsticks. Samples were rinsed in buffered peptone water for bacteria isolation. Over six log CFU/sample of aerobic plate counts (APC), lactic acid bacteria, and coliforms were detected on post-pick samples. All spoilage bacteria were reduced to nondetectable levels on post-chill samples (*p* < 0.001). However, the presence of all bacteria on mechanically deboned meat (MDM) samples indicated varying degrees of cross contamination from post-chill and MDM samples. These results suggest PAA effectively reduces spoilage microbes in chilling applications irrespective of differences in PAA concentrations. However, due to the levels of spoilage microbes detected in MDM, it may be worth investigating the potential interventions for this stage of processing.

## 1. Introduction

Spoilage microbes are commonly found in processing and although they do not present a major health burden, they do present economic challenges to the industry. Microbial spoilage is a critical factor limiting broiler meat shelf life [1]. Spoilage leads to biochemical alterations in broiler meat, which can affect sensory characteristics, such as color and odor [2]. A multitude of studies have reported the deleterious effects of spoilage microbes on broiler meat [2,3,4]. In contrast to *Campylobacter*, *Salmonella*, and other pathogenic microbes, spoilage bacteria specifically in poultry processing are studied much less. Most of the literature is centered around spoilage bacteria behavior in different storage conditions [5]. The literature involving spoilage bacteria in post-processing primarily investigates total aerobic bacterial counts, lactic acid bacteria, coliforms, and even *Pseudomonas*. By determining the aerobic plate counts (APC), a general measurement of all microbial growth on broiler meat can be obtained [6]. Coliforms are another group of spoilage microbes, which impact broiler meat shelf life, as they are an indication of fecal contamination on meat [7]. Lactic acid bacteria (LAB) are microbes more prevalent in modified-atmospheric-packaged and vacuum-packaged meat products [8]. Lastly, *Pseudomonas* are psychrotrophic bacteria commonly found on meat stored under refrigeration [9]. The presence of high initial-spoilage microbial loads can lead to a shortened shelf life of products and lead to deleterious effects on taste, appearance, and odor [5]. Therefore, it is important to have proper antimicrobial interventions to reduce the spoilage microbial load and to enhance the shelf life and quality of poultry products. Moreover, as the last phase before retail distribution, broiler processing has the capability of greatly affecting meat sensory characteristics and shelf-life potential by microbial reduction treatments.

Food Safety Inspection Services (FSIS) use strict performance standards in poultry processing plants to keep contamination levels of pathogenic bacteria, such as *Salmonella* and *Campylobacter*, below specific percentages throughout a 52-week period [10]. Currently, there is no such performance standard for spoilage microbes, as they do not present a major food safety concern. However, antimicrobial treatments commonly used to reduce pathogenic microbial prevalence are also effective against common spoilage bacteria [11]. Currently, the antimicrobial most commonly employed in poultry processing is peracetic acid (PAA). According to USDA Safe and Suitable Ingredients standards, PAA has a maximum permissible limit of 2000 ppm and is applied in spray and immersion-chilling applications [12].

Although various studies have reported the effects of PAA on spoilage growth during meat storage, there are few studies comparing the efficacy of PAA applications against spoilage microbes during processing between multiple processing plants. Moreover, limited data are available on the change in spoilage prevalence between processing steps in commercial processing plants using PAA. However, determining the initial spoilage bacteria incidence may provide a more accurate analysis of the effects of PAA during processing and help provide correlations between initial spoilage counts and shelf stability of broiler meat products.

Therefore, the objective of this study was to determine the prevalence of total aerobic bacteria, coliforms, lactic acid bacteria, and *Pseudomonas* at five stages of poultry processing in commercial plants that use different concentrations of PAA.

## 2. Materials and Methods

### 2.1. Experimental Design

Three commercial broiler processing plants that use peracetic acid as a primary antimicrobial intervention were selected for this study. All three plants were operated by the same integrator and were managed with similar standard operating procedures. While samples were collected at the same processing steps in each plant, there were minor logistical differences in the layout due to differences in plant design. At each processing plant, ten broiler meat samples were collected from five processing steps (per replication x three): post-pick, pre-chill, post-chill, mechanically deboned meat (MDM), and drumsticks. Each visit to the processing plant was considered a replication. One of the three plants did not mechanically debone meat. Therefore, the prevalence of microbes on MDM was analyzed independently. A total of 420 samples were collected. Each plant utilized peracetic acid as the primary antimicrobial in similar applications. However, there were logistical differences between treatments and differences in the concentration of PAA applied between the plants. All three plants used online-reprocessing (OLR) cabinets for pre-chill carcasses, pre-chiller, drag-chiller, and finishing chiller tanks for post-chill carcasses, and dip tanks for drumsticks. Plant one had an additional intervention step for post-pick carcasses: New York rinse cabinets. For all three plants, there was no intervention for MDM samples. At each sampling, the concentrations of PAA from application at each processing step were recorded.

### 2.2. Sampling

Samples were collected by using sterile gloves to transfer carcasses, MDM, and drumsticks into 15 in × 20 in 3M sterile bird rinse bags. Carcasses were rinsed at the processing plant with 400 mL of buffered peptone water (BPW) (3M, Saint Paul, MN, USA) for one minute. The rinsate was poured back into the 3M bottles, the carcasses were returned to the rehang line, and then, the rinsate was stored on ice with the drumsticks and MDM for 1–3 h during transit back to the Mississippi State University Poultry Science BSL-2 laboratory. Upon arrival, drumsticks and MDM were enriched in 3M buffered peptone water (BPW) as per USDA isolation guidelines MLG 4.10 [13]. Drumsticks were rinsed in 225 mL of BPW for one minute. Mechanically deboned meat was aliquoted into 25 g samples using sterile weigh boats and a scale. Each sample was transferred to a sterile Whirl-Pak bag (Nasco Sampling/Whirl-Pak^®^, Madison, WI, USA) and rinsed with 225 mL of BPW for 1 min. Serial dilutions were prepared using BPW rinsate, and these dilutions were used for spoilage bacteria enumeration.

### 2.3. Bacteria Isolation and Enumeration

Prior to plating for each bacterium, rinsate from each sample was serially diluted in BPW. Total aerobic bacteria were enumerated by directly plating 100 µL of broiler meat rinsate onto standard plate count agar via the spread plate method. The plates were incubated under aerobic conditions at 37 °C for 24 h. Lactic acid bacteria were enumerated by directly plating 100 µL of rinsate dilutions onto De Man, Rogosa, Sharpe (MRS) agar (Oxoid, Thermo Fisher Scientific, Waltham, MA USA), and the plates were incubated at 37 °C for 48 h in anaerobic conditions using a Spiral Biotech ANOXOMAT (Spiral Biotech, Norwood, MA, USA). Coliforms were enumerated by pipetting 1mL of rinsate dilutions onto 3M coliform petrifilms (3M, Saint Paul, MN, USA), and the petrifilms were incubated at 37 °C for 24–48 h in aerobic conditions. *Pseudomonas* were enumerated by directly plating 100 µL of rinsate dilutions onto cetrimide agar, and plates were incubated at 37 °C for 24–48 h in aerobic conditions. Colonies that turned green or demonstrated fluorescence were deemed positive as *Pseudomonas*.

### 2.4. Statistical Analysis

Data were analyzed as a completely randomized design with a 3 × 4 factorial arrangement of treatments (3 processing plants and 4 processing steps). Microbial counts of MDM were analyzed separately using Student’s t-test to determine the differences among processing plants. Analysis of variance was performed by the GLIMMIX procedure of SAS version 9.4 (SAS Institute Inc., Cary, NC, USA). Means were separated by protected t-test in the LSMEANS procedure. Statistical significance was determined at a *p* value of ≤ 0.05.

## 3. Results

### 3.1. Aerobic Plate Counts

**Figure 1:**
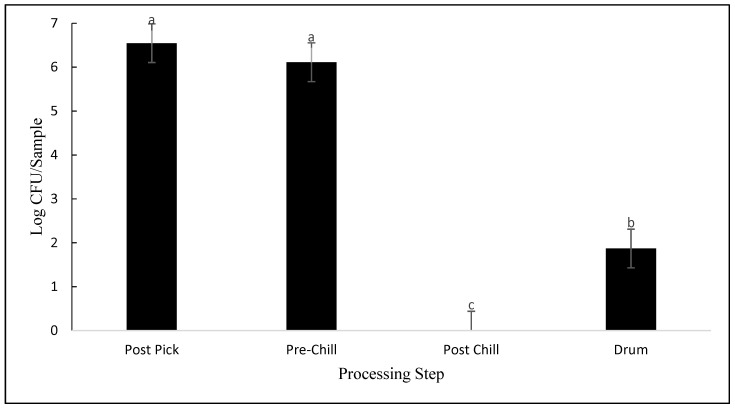
APC contamination expressed as log CFU/sample, detected from samples collected at post-pick, pre-chill, post-chill, and drumsticks (*p* < 0.001). Means with different letters differ statistically.

**Figure 2:**
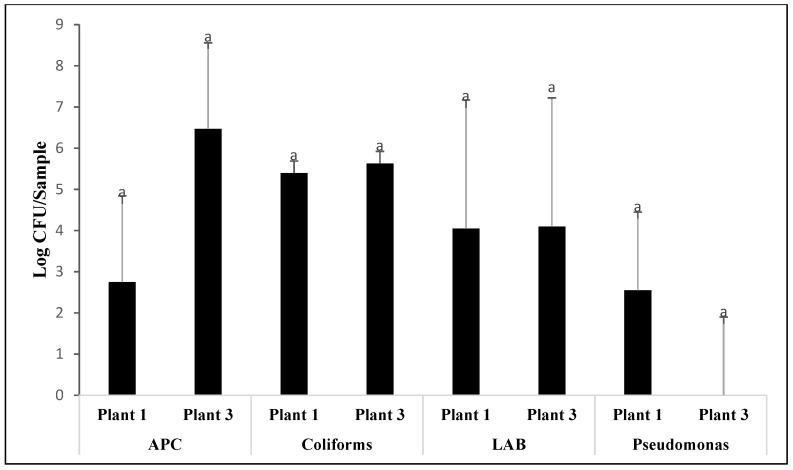
Aerobic plate count (APC), coliforms, lactic acid bacteria (LAB), and *Pseudomonas* contamination expressed as log CFU/sample, detected from MDM samples in processing plants 1 and 3 (*p* = 0.174). Means with different letters differ statistically.

The only differences observed for APC were between the processing steps (*p* < 0.001). As seen in Figure 1, there were no significant differences between post-pick and pre-chill, with carcass contamination averaging 6.5 log CFU/sample and 6.1 log CFU/sample, respectively. However, in post-chill samples, APCs were reduced to nondetectable levels (*p* < 0.001), a 6.1 log CFU/sample reduction. Drumsticks had slightly higher APCs, averaging 1.87 log CFU/sample, as compared to post-chill carcasses (*p* = 0.005). In MDM samples, there was no statistically significant difference in APCs between plants 1 (2.75 log CFU/sample) and 3 (6.4 log CFU/sample; *p* = 0.174; Figure 2).

### 3.2. Coliforms

**Figure 3:**
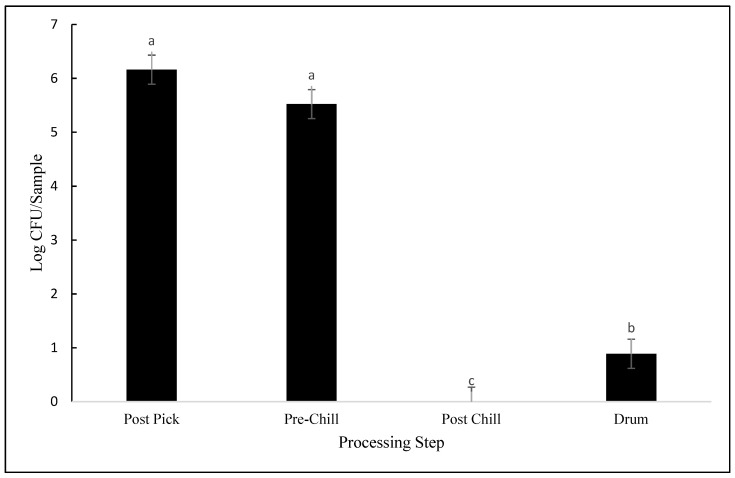
Coliform contamination expressed as log CFU/sample, detected from samples collected at post-pick, pre-chill, post-chill, and drumsticks (*p* < 0.001). Means with different letters differ statistically.

Differences between the processing steps were observed for coliform contamination as well (*p* < 0.001; Figure 3). No statistically significant differences were observed between post-pick and pre-chill, with contamination levels averaging 6.16 log CFU/sample and 5.52 log CFU/sample, respectively (*p* = 0.109). However, coliform counts were reduced to nondetectable levels in post-chill carcasses (*p* < 0.001). While coliforms were undetectable in post-chill samples, the counts were greater in drumsticks, with the average coliform counts reaching 0.89 log CFU/sample. As seen in Figure 2, there were no differences between plants 1 and 3 in MDM coliform counts levels, averaging 5.4 log CFU/sample and 5.6 log CFU/sample, respectively (*p* = 0.506).

### 3.3. Lactic Acid Bacteria

**Figure 4:**
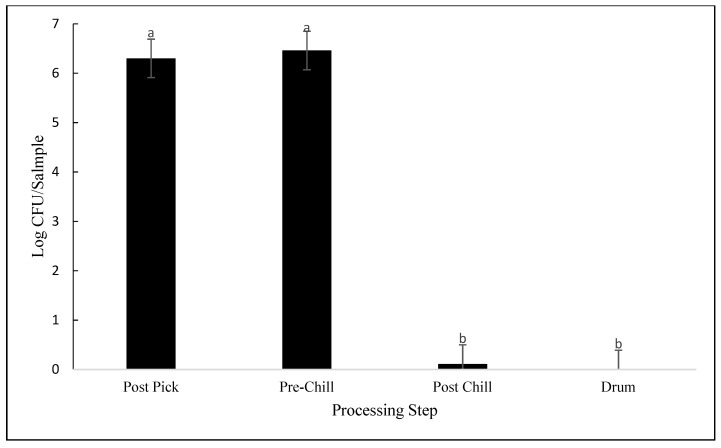
Lactic acid bacteria contamination expressed as log CFU/sample, detected from samples collected at post-pick, pre-chill, post-chill, and drumsticks (*p* < 0.001). Means with different letters differ statistically.

No differences were observed in LAB counts between the processing plants (*p* = 0.681). Only the processing step influenced LAB counts (*p* < 0.001). As seen in Figure 4, there was no difference in LAB counts between post-pick and pre-chill carcasses, with average contamination levels being 6.30 log CFU/sample and 6.46 log CFU/sample, respectively (*p* = 0.766). However, there was a significant difference between pre-chill and post-chill (*p* < 0.001). In post-chill samples, lactic acid bacteria were reduced by 6.35 log CFU/sample to nondetectable levels. Lactic acid bacteria were nondetectable in drumsticks. There were no significant differences in MDM contamination between plants 1 and 3 (*p* = 0.988). As seen in Figure 2, lactic acid bacteria contamination in MDM averaged 4.05 log CFU/sample and 4.1 log CFU/sample for plants 1 and 3, respectively.

### 3.4. Pseudomonas

**Figure 5:**
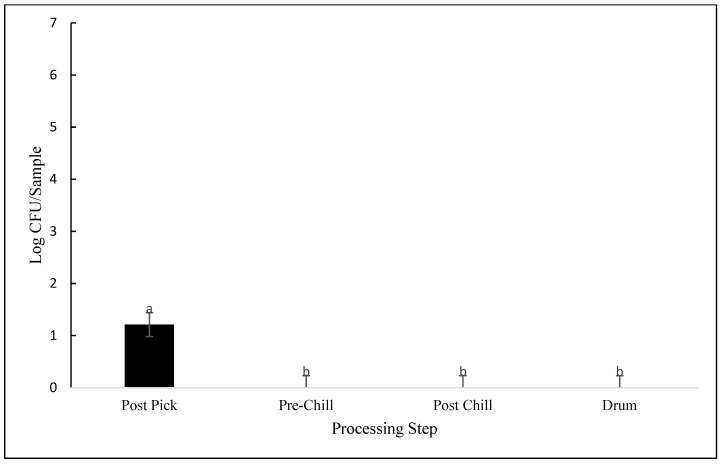
*Pseudomonas* contamination expressed as log CFU/sample, detected from samples collected at post-pick, pre-chill, post-chill, and drumsticks (*p* = 0.001). Means with different letters differ statistically.

Differences between processing plants and processing steps were observed for *Pseudomonas* counts (*p* < 0.001). As seen in Figure 5, *Pseudomonas* were only detected in plant 2 during post-pick sampling, with contamination levels averaging 1.21 log CFU/sample. Although not detected in the other three steps analyzed, *Pseudomonas* were found in MDM samples of plant 1. As seen in Figure 2, MDM samples in plant 1 were contaminated with an average of 2.55 log CFU/sample, whereas no *Pseudomonas* were detected in MDM in plant 3. However, this difference was not considered statistically significant (*p* = 0.272).

## 4. Discussion

### 4.1. Aerobic Bacteria Contamination

Based on the results, APC concentrations were reduced in all three processing plants during processing. The initial carcass contamination levels in post-pick samples in this study were similar to previous findings [14]. In post-pick samples in this study, APC contamination averaged 6.5 log CFU/sample, whereas Zhang found an average of 6.45–6.64 log CFU/g. However, no significant differences were seen between post-pick and pre-chill samples in the study. APC concentrations were only reduced by 0.4 log CFU/sample in this study, whereas Zhang reported reductions of 2.4–2.5 log CFU/g [14]. Similarly, other research has reported similar reductions in APC concentrations on pre-chill/post-evisceration samples. While APC concentrations in this study were found to be an average of 6.1 log CFU/sample, Zhang reported concentrations of 2.98 log CFU/g [15]. One study with similar peracetic acid treatments also found lower APC populations of around 3.59 log CFU/mL in pre-chill samples [16]. Virtually no studies in the last 20 years reported total aerobic bacteria concentrations on broiler cut-up parts, such as drumsticks. Only one recent publication mentions an increase in spoilage bacteria contamination on cut-up parts, as observed in this study [16]. Although data were not collected directly from processing, one shelf-life study found APC contamination on drumsticks to be an average of 5 log CFU/g on the day of packaging [17]. This was much greater than the APC contamination found in this study (1.87 log CFU/sample).

### 4.2. Coliform Contamination

The initial coliform contamination in post-pick carcasses was similar to other bacteria in this study. Pre-chill and post-chill coliform contamination in this study was similar to previous findings. Coliform populations were reported to be 3 log CFU/mL in pre-chill carcasses and 0.17 log CFU/mL in post-chill carcasses [18], whereas in this study, pre-chill contamination averaged 5.2 log CFU/sample and was reduced to nondetectable levels in post-chill samples. Contamination in drumsticks averaged 0.89 log CFU/sample, whereas another study found drumstick contamination to be an average of 5.6 log CFU/sample [19]. However, drumsticks used by Smith (2010) were cut from carcasses that had not been immersed in chilling tanks.

### 4.3. Lactic Acid Bacteria Contamination

Based on the results from this study, lactic acid bacteria concentrations were reduced throughout the processing chain in all three plants. However, there was an increase in prevalence seen in MDM. Concentrations of lactic acid bacteria in post-pick samples in this study differed from previous findings [20]. In this study, the initial concentrations were much higher, at 6.3 log CFU/sample, whereas previously, concentrations were reported at 3.8 log CFU/mL [20]. However, LAB was nondetectable in drumsticks in this study. There is limited research reporting the populations of LAB in drumsticks during processing. However, one study looked at the populations of LAB in marinated drumsticks during refrigeration. Although not directly comparable, the initial concentrations of LAB on the first day of storage were 2.9 log CFU/g [21]. By day 17, the concentrations were similar to initial contamination levels found in this study.

### 4.4. Pseudomonas Contamination

What was most surprising was the lack of *Pseudomonas* found in samples from all three plants. Historically, *Pseudomonas* have been highly prevalent in broiler processing [22,23,24]. However, based on previous findings by Hinton jr. and Ingram, 2004, Wang et al., 2017, Wang et al., 2019, and Wages et al., 2019, a trend demonstrating lower recovered levels of *Pseudomonas* over the last 10 years can be seen [25,26,27,28]. Hinton Jr. and Ingram’s early findings in 2004 found 3 log CFU/mL of *Pseudomonas* in broiler carcasses at early stages of processing. However, by 2019, a lack of *Pseudomonas* in processing was reported from multiple studies [28]. This may in part be due to shifts in the commercial standard to use PAA as the primary antimicrobial, which is known to exhibit significant toxicogenomic effects against *Pseudomonas* [29].

### 4.5. MDM Contamination

Significant levels of APC, lactic acid bacteria, and coliforms were found in MDM samples. These findings were consistent with previous research, which suggests the increased surface contact from mechanically deboning meat accounts for higher rates of cross contamination [5,30]. There is currently a lack of research pertaining to contamination levels in MDM samples. However, a more recent experiment found similar APC contamination in broiler MDM at an average of 4 log CFU/sample [31], whereas in this study, APC concentrations in MDM samples ranged from 2.75 to 6.4 log CFU/sample. Although there were differences in the bacteria detected in this study, previous research has found broiler MDM contaminated with *Escherichia coli*, *Staphylococcus aureus*, *Campylobacter*, and *Salmonella* [31]. These findings are congruent with findings in this study, which isolated multiple microbes from broiler MDM. Coliform contamination specifically in MDM samples was noticeably higher than other microbes analyzed during this step. Contamination levels in MDM closely resembled initial coliform levels in post-pick samples, which suggests alarming rates of cross contamination during MDM processing. In all three plants, there was no antimicrobial intervention for MDM samples. Due to the levels of contamination in MDM, an intervention method may be worth investigating, as high spoilage bacteria prevalence may be indicative of a higher prevalence of pathogenic foodborne pathogens. High initial spoilage counts can lead to a shorter shelf life of meat products, thus contributing to greater profit losses and food waste [32]. As pointed out by previous literature, spoilage is a multifactorial process dependent upon the types of microorganisms and environmental conditions of the broilers [33]. Although spoilage is not inherently correlated with total viable counts, having high concentrations of multiple spoilage microbes could trigger early meat spoilage before the expected product expiration dates, leading to great financial loss [33].

### 4.6. Peracetic Acid

Peracetic acid was the primary antimicrobial utilized in all three processing plants sampled in this study. In Table 1, the average PAA concentrations applied at each step from the three plants can be seen. Based on the results, PAA reduced the microbial populations of all bacteria in this study. However, similar results were seen in all three plants, irrespective of PAA concentration. Although plant 1 utilized New York Rinse cabinets with PAA on post-pick samples, the microbial load did not differ from the other plants that had no antimicrobial treatment at this step. As there were no major differences in the microbial load between the processing plants, these results would indicate that the inclusion of first-processing PAA spray cabinets is ineffective. The greatest variation in PAA concentration was observed in the carcass chilling tanks. Despite these differences, all tested spoilage microbes were reduced to nondetectable levels in post-chill samples. As there were no major statistical differences observed, this would suggest that the lowest effective concentration could be utilized in chilling tanks. Although there is a limited number of publications investigating the effects of PAA on spoilage microbes, the efficacy of PAA applications in carcass chilling tanks has been well established [34,35,36,37]. Based on the findings in this study, as well as previous literature, peracetic acid in carcass chilling tanks is still the most effective antimicrobial intervention in commercial poultry processing.

## 5. Conclusions

The results from this study indicate that, overall, PAA was effective at reducing the prevalence of all bacteria analyzed in this study, irrespective of the processing plant. However, compared to the other steps, the carcass chillers were much more effective at reducing the bacteria observed in this study. Based on these findings, we would recommend using the lowest effective concentration of PAA in finishing chillers, as it could improve economic savings and preserve surface proteins in meat, allowing for better sensory characteristics. Significant differences between the microbial load of post-chill and MDM samples suggest high levels of cross contamination occur during MDM, and an intervention may be worth investigating for this step. A higher prevalence of APC and coliforms in drumsticks also suggests cross contamination during deboning, which could impact the shelf life of packaged chicken parts, similar to drumsticks.

## Figures and Tables

**Table 1:** Description of PAA concentrations (ppm) used at each step. Concentrations with the greatest impact on microbial levels are in bold.

Antimicrobial Intervention	Plant 1	Plant 2	Plant 3
NY rinse cabinets	183		
OLR Cabinets	187	143	138
Pre-Chiller	35	76	38
Drag chiller	23	75	38
Finishing chiller	767	412	705
Dip tank	670	430	480

## Data Availability

The data presented in this study are available on request from the corresponding author.
